# Occupational stress and associated factors among general practitioners in China: a national cross-sectional study

**DOI:** 10.1186/s12889-022-13484-3

**Published:** 2022-05-27

**Authors:** Jing Feng, Heng Jiang, Xin Shen, Zihui Lei, Liqing Li, Yi Zhu, Mingye Zhang, Tingting Yang, Xin Meng, Hongkun Di, Wenqi Xia, Zuxun Lu, Yong Gan

**Affiliations:** 1 Department of Social Medicine and Health Management, School of Public Health, Tongji Medical College, Huazhong University of Science and Technology, Wuhan, Hubei China; 2 Centre for Alcohol Policy Research, School of Psychology and Public Health, La Trobe University, Melbourne, Victoria Australia; 3 Centre for Health Equity, Melbourne School of Population and Global Health, University of Melbourne, Melbourne, Victoria Australia; 4 Department of Management Science and Engineer, School of Economics and Management, Jiangxi Science and Technology Normal University, Nanchang, Jiangxi China; 5 Department of Nutrition, Henan Provincial People’s Hospital and Zhengzhou University People’s Hospital, Zhengzhou, Henan China

**Keywords:** General practitioners, Occupational stress, Primary health care, China

## Abstract

**Background:**

Occupational stress among general practitioners (GPs) is a public health concern. This study aimed to investigate the prevalence and factors associated with occupational stress among GPs in China.

**Methods:**

A cross-sectional design was used. Data were collected from 3,236 GPs in eastern, central, and western China (response rate, 99.75%) between October 2017 and February 2018 using a structured self-administered questionnaire. An ordinal logistic regression model was used to identify the factors associated with occupational stress among GPs.

**Results:**

Among these respondents, 313 (9.67%), 1,028 (31.77%), and 1,895 (58.56%) of GPs had a low, medium, and high level of occupational stress, respectively. GPs from central China, with temporary work contracts, without management responsibility, receiving a moderate level of income, and with moderate occupational development opportunities had a lower level of occupational stress. GPs with greater than 40 working hours per week and those who worked overtime occasionally or frequently had a higher level of occupational stress.

**Conclusions:**

The prevalence of occupational stress among GPs is high in China. Substantial regional variation in determinants of occupational stress among GPs was observed. These findings should inform the design of policies to reduce the occupational stress of GPs.

## Background

Occupational stress, defined as the worker’s physical and emotional response to occupational demands exceeding their capacity, is a major occupational health hazard [1]. Occupational stress is linked to a wide variety of adverse health outcomes, including stroke [2], cardiovascular disease [3], anxiety [4], and depression [5]. It may also adversely impact organizations through its contribution to absenteeism [6], staff turnover [6, 7], and workforce shortages [8]. Healthcare workers experience relatively high levels of occupational stress when compared to workers in other sectors. This is especially true of general practitioners (GPs), a group ranking highly with respect to occupational stress among the healthcare professions [9]. GPs are usually a person’s first point of contact with the healthcare system and they are key in the prevention, monitoring and management of a variety of health concerns. Anything affecting the performance of a nation’s GP workforce will subsequently impact the quality of the nation’s wider healthcare system. Therefore, the primary prevention of occupational stress in GPs should be considered a public health priority.

The Global Conference on Primary Health Care 2018 held in Astana, Kazakhstan, designated the improvement of primary healthcare a current global public health priority. The Chinese government outlined their goal of improving the capability of the primary health system in the “Healthy China 2030” plan, but at present, community healthcare institutions in China are experiencing GP shortages and high GP turnover. One possible contributor to these issues in the GP workforce is occupational stress [10]. Moderating occupational stress is key in the administration of healthcare services, but this is not possible without first identifying the factors that contribute to occupational stress in any given context. Studies investigating GP occupational stress have been conducted in many countries [11–19], but to date, no research on occupational stress and its determinants has been conducted among Chinese GPs at the national level. This study aimed to address this research gap. The results of this study will contribute to the improvement of existing strategies to reduce GP occupational stress in China and provide valuable evidence on the topic for the international general practice research field.

## Methods

### Study population

A national cross-sectional study was conducted between October 2017 and February 2018 in China. A multi-stage stratified random sampling strategy was used. Four provinces were randomly selected from each of eastern (Shanghai, Beijing, Guangdong, and Zhejiang), central (Hubei, Anhui, Heilongjiang, and Henan), and western (Sichuan, Chongqing, Guizhou, and Yunnan) China; within each of the 12 selected provinces, we randomly selected 30 community health service institutions; within each selected institution, we randomly selected 40% of on-post GPs with ≥ 1 year work experience for study inclusion. A total of 3,244 GPs were invited through WeChat to complete a self-administered questionnaire. Among them, only 8 GPs did not respond, meaning 3,236 responses were eligible for the analysis (yielding a response rate of 99.75%). The collected data were used in a previous article published in 2020 [20].

The study protocol was approved by the Ethics Committee of the Tongji Medical College Institutional Review Board, Huazhong University of Science and Technology, Wuhan, China (no. [2018] IEC (S186)). Informed consent was obtained from all survey participants.

### Instrument and measurement

The questionnaire comprised six parts: socio-demographic information, occupational stress, job satisfaction, professional identity, burnout, and turnover intention. Given the purpose of this study, the data from sections 1 and 2 were included. Socio-demographic data items were region, age, gender, marital status, education level, work tenure, contract status, professional title, management responsibility, income level, working hours per week, working overtime, and occupational development opportunities. Although some occupational stress scales have been reported in the literature, no specific questionnaire was available for GPs in China. Therefore, occupational stress was evaluated with the question: “How stressful is your job as a GP?” and was measured on a 5-point Likert scale: not at all stressful; somewhat stressful; moderately stressful; very stressful; extremely stressful. On the basis of a literature review and group discussions, we designed one closed-ended, multiple-choice question with 7 response options [economic pressure (low wages or the pressure of buying a house), having difficulties achieving assessment targets, lacking professional identity, time pressure (lacking time to care for parents or children), promotion pressure, self-health status, and lacking attention from leadership] to assess the source of occupational stress.

### Data collection and quality control

The questionnaire was designed based on a literature review, group discussions, and mock interviews. We had invited 20 healthcare experts from China to evaluate the content validity of the measure. In addition, a pretest involving 40 GPs was conducted in Wuhan’s community health centers (CHCs) to improve the quality of the questionnaire. A total of 38 of those respondents were able to clearly understand all of the contexts of the questionnaire, and further modifications were made according to their feedback. This showed that the questionnaire had a good content validity. Community Health Association of China organized and carried out the survey. A web link to the online questionnaire designed using the software Questionnaire Star was disseminated to the GPs through WeChat. GPs completed the questionnaire on a voluntary basis, and all participants provided written or verbal informed consent before participating in the study. In addition, we offered incentives for the participants, called “Chinese WeChat Awarding Lucky Red Bag”. Importantly, Chinese GPs were honest and loyal to the Community Health Association of China, and they deeply understood the issues in the current primary healthcare system and were willing to improve the current situation by participating in this survey. Thus, a high response rate was achieved. Data were automatically collected by Questionnaire Star through the WeChat platform. A few text formats of data (including ethnicity, region, names of primary healthcare institutions, etc.) were encoded and all data were entered into the Web-based database by trained investigators to ensure accuracy.

### Data analysis

All analyses were performed using Statistical Package for Social Sciences (SPSS, Inc., Chicago, IL, Version 13.0). Chi-square tests were conducted to compare occupational stress between categories. An ordinal logistic regression model was used to identify risk factors of occupational stress among GPs. Occupational stress, the dependent variable, was treated as a variable with three categories. “Not at all stressful” and “somewhat stressful” were classified as “low level of occupational stress”. “Moderately stressful” represented “medium level of occupational stress”. “Very stressful” and “extremely stressful” were classified as “high level of occupational stress”. In the ordinal logistic regression model, predictive variables included all characteristics of GPs. In addition, a stratified analysis was used to investigate the determinants of occupational stress among GPs across regions. The proportional odds assumption was supported by the Brant test of the parallel regression assumption and the likelihood-ratio test of the proportionality of odds across response categories. A value of *P* < 0.05 (two-tailed) was considered statistically significant.

## Results

The characteristics of respondents are presented in Table 1. Among the 3,236 GPs, 313 (9.67%), 1,028 (31.77%), and 1,895 (58.56%) had a low, medium, and high level of occupational stress, respectively. The mean age of participants was 37.42 years [standard deviation (SD) = 7.92] and more than half (63.84%) were females. Participants from eastern, central, and western China numbered 1,229 (37.98%), 971 (30.01%), and 1,036 (32.01%), respectively. Most respondents (85.63%) were married and 2,139 (66.10%) GPs had a bachelor’s degree. The mean tenure of GPs was 7.29 years (SD = 5.94) and more than half (67.52%) had permanent work contracts. Only 12.52% GPs had senior professional titles. Most participants (75.83%) had no management responsibilities. Most GPs (70.49%) had a low level of income. More than half (58.16%) of respondents reported working less than 40 h per week while few respondents (5.62%) reported working overtime. Few participants (7.32%) reported having high level of occupational development opportunities.

**Table 1:** Descriptive statistics and univariate analysis of the differences of occupational stress among GPs

Variables	Frequency (%)	Low (%)	Moderate (%)	High (%)	*χ*^*2*^	*P* value
**Total**	3236 (100.00)	313 (9.67)	1028 (31.77)	1895 (58.56)		
**Region**						
Eastern China	1229 (37.98)	94 (7.65)	384 (31.24)	751 (61.11)	16.00	< 0.01
Central China	971 (30.01)	121 (12.46)	309 (31.82)	541 (55.72)		
Western China	1036 (32.01)	98 (9.46)	335 (32.34)	603 (58.20)		
**Age (years)**						
21–	515 (15.91)	62 (12.04)	218 (42.33)	235 (45.63)	54.81	< 0.01
30–	1454 (44.93)	131 (9.01)	464 (31.91)	859 (59.08)		
40–	1063 (32.85)	94 (8.84)	283 (26.62)	686 (64.53)		
50–	204 (6.30)	26 (12.75)	63 (30.88)	115 (56.37)		
**Gender**						
Male	1170 (36.16)	91 (7.78)	315 (26.92)	764 (65.30)	34.55	< 0.01
Female	2066 (63.84)	222 (10.75)	713 (34.51)	1131 (54.74)		
**Marital status**						
Unmarried/widowed/divorced	465 (14.37)	55 (11.83)	186 (40.00)	224 (48.17)	24.20	< 0.01
Married	2771 (85.63)	258 (9.31)	842 (30.39)	1671 (60.30)		
**Education level**						
Associate’s degree or vocational diploma^a^	918 (28.37)	100 (10.89)	301 (32.79)	517 (56.32)	11.40	0.02
Bachelor degree	2139 (66.10)	186 (8.70)	672 (31.42)	1281 (59.89)		
Master degree or higher	179 (5.53)	27 (15.08)	55 (30.73)	97 (54.19)		
**Work tenure (years)**						
1–	2241 (69.25)	230 (10.26)	758 (33.82)	1253 (55.91)	22.66	< 0.01
10–	825 (25.49)	69 (8.36)	217 (26.30)	539 (65.33)		
20–	170 (5.25)	14 (8.24)	53 (31.18)	103 (60.59)		
**Contract status**						
Permanent	2185 (67.52)	194 (8.88)	651 (29.79)	1340 (61.33)	21.43	< 0.01
Temporary	1051 (24.38)	119 (11.32)	377 (35.87)	555 (52.81)		
**Professional title**						
Elementary or below	1419 (43.85)	154 (10.85)	515 (36.29)	750 (52.85)	38.84	< 0.01
Intermediate	1412 (43.63)	114 (8.07)	412 (29.18)	886 (62.75)		
Senior	405 (12.52)	45 (11.11)	101 (24.94)	259 (63.95)		
**Management responsibility**						
Yes	782 (24.17)	46 (5.88)	149 (19.05)	587 (75.06)	115.75	< 0.01
No	2454 (75.83)	267 (10.88)	879 (35.82)	1308 (53.30)		
**Income level**						
Low	2281 (70.49)	210 (9.21)	726 (31.83)	1345 (58.97)	4.62	0.33
Moderate	864 (26.70)	93 (10.76)	280 (32.41)	491 (56.83)		
High	91 (2.81)	10 (10.99)	22 (24.18)	59 (64.84)		
**Working hours per week**						
≤ 40	1882 (58.16)	241 (12.81)	725 (38.52)	916 (48.67)	185.36	< 0.01
> 40	1354 (41.84)	72 (5.32)	303 (22.38)	979 (72.30)		
**Working overtime**						
Never	182 (5.62)	54 (29.67)	79 (43.41)	49 (26.92)	360.55	< 0.01
Occasion	1759 (54.36)	209 (11.88)	694 (39.45)	856 (48.66)		
Frequent	1295 (40.02)	50 (3.86)	255 (19.69)	990 (76.45)		
**Occupational development opportunities**						
Low	1650 (50.99)	153 (9.27)	470 (28.48)	1027 (62.24)	28.70	< 0.01
Moderate	1349 (41.69)	131 (9.71)	494 (36.62)	724 (53.67)		
High	237 (7.32)	29 (12.24)	64 (27.00)	144 (60.76)		

*Abbreviations: GPs* general practitioners

^a^GPs who have acquired associate degree or vocational diploma. An associate degree required 3 years of education in college after graduation from senior middle school (grade year 10 to year 12), or 5 years of education in college after graduation from junior middle school (grade year 7 to year 9). A vocational diploma requires 2 years of education in vocational schools after graduation from senior middle school, or 3 years of education in vocational schools after graduation from junior middle school

Table 1 shows the differences in occupational stress for various groups. Significant differences in GPs’ occupational stress were found across regions, ages, genders, marital statuses, education levels, work tenures, contract statuses, professional titles, management responsibilities, levels of working hours per week, working overtime, and occupational development opportunities (*P* < 0.05). There were no significant differences in occupational stress among income levels (*P* > 0.05). When we investigated the source of occupational stress, economic pressure appeared to be the main stressor for most GPs (76.41%) (Table 2).

**Table 2:** Distribution of the source of occupational stress among GPs

Items	N	%
Economic pressure (low wages or the pressure of buying a house)	2445	76.41
Having difficulties achieving assessment targets	1366	42.69
Lacking professional identity	1341	41.91
Time pressure (Lacking time to care for parents or children)	1272	39.75
Promotion pressure	1003	31.34
Self-health status	684	21.38
Lacking attention from leadership	624	19.50

*Abbreviations:GPs* general practitioners

Table 3 shows the results from the ordinal logistic regression analysis to determine factors associated with GPs’ occupational stress. Region, contract status, management responsibility, income level, working hours per week, working overtime, and occupational development opportunities were significantly associated with occupational stress. GPs from central China [odds ratio (OR) = 0.81, 95% CI: 0.67–0.98)], who had a temporary work contract (OR = 0.84, 95% CI: 0.71–1.00), who had no management responsibility (OR = 0.44, 95% CI: 0.35–0.54), who were at a moderate level of income (OR = 0.77, 95% CI: 0.65–0.92) or who had moderate occupational development opportunities (OR = 0.79, 95% CI: 0.67–0.92) had lower occupational stress. GPs with working hours greater than 40 h per week (OR = 1.89, 95% CI: 1.61–2.23) and those who worked overtime occasionally (OR = 2.50, 95% CI: 1.86–3.35) or frequently (OR = 6.52, 95% CI: 4.76–8.93) had a higher level of occupational stress.

**Table 3:** Ordinal logistic regression model analysis for the association with the occupational stress among GPs

**Variables**	**National**^a^			**Eastern China**^**b**^			**Central China**^**b**^			**Western China**^**b**^		
	**OR**	**95% CI**	***P value***	**OR**	**95% CI**	***P value***	**OR**	**95% CI**	***P value***	**OR**	**95% CI**	***P value***
**Region (ref: eastern China)**												
Central China	0.81	0.67–0.98	0.028									
Western China	0.90	0.75–1.09	0.296									
**Age (ref: 21-)**												
30-	1.11	0.87–1.41	0.393	1.40	0.90–2.17	0.132	1.03	0.67–1.59	0.885	1.10	0.74–1.63	0.649
40-	1.07	0.80–1.42	0.670	1.48	0.88–2.50	0.144	1.03	0.61–1.73	0.918	0.96	0.59–1.57	0.879
50-	0.75	0.50–1.13	0.171	1.21	0.59–2.49	0.608	0.80	0.39–1.65	0.551	0.43	0.20–0.89	0.023
**Gender (ref: male)**												
Female	0.94	0.80–1.10	0.431	0.97	0.75–1.26	0.821	0.75	0.57–0.99	0.045	1.19	0.88–1.61	0.267
**Marital status (ref: unmarried/widowed/divorced)**												
Married	1.11	0.89–1.39	0.350	1.29	0.87–1.90	0.200	0.82	0.54–1.22	0.322	1.28	0.87–1.86	0.206
**Education level (ref: associate’s degree or vocational diploma)**												
Bachelor degree	1.06	0.88–1.27	0.557	1.12	0.76–1.66	0.565	1.15	0.83–1.58	0.402	0.98	0.74–1.31	0.894
Master degree or higher	0.82	0.58–1.17	0.277	0.91	0.51–1.61	0.739	0.86	0.45–1.67	0.663	0.71	0.32–1.58	0.397
**Work tenure (years, ref: 1-)**												
10-	1.14	0.94–1.37	0.192	1.05	0.78–1.42	0.734	1.18	0.83–1.67	0.364	1.18	0.81–1.72	0.389
20-	0.90	0.63–1.28	0.556	0.77	0.43–1.37	0.378	0.99	0.53–1.83	0.969	0.85	0.43–1.68	0.647
**Contract status (ref: permanent)**												
Temporary	0.84	0.71–1.00	0.047	0.99	0.69–1.41	0.950	0.90	0.67–1.21	0.502	0.71	0.54–0.94	0.017
**Professional title (ref: elementary or below)**												
Intermediate	1.16	0.96–1.39	0.121	0.97	0.70–1.34	0.852	1.31	0.94–1.82	0.108	1.11	0.80–1.54	0.545
Senior	0.89	0.65–1.20	0.443	0.54	0.33–0.91	0.020	0.90	0.52–1.57	0.711	1.45	0.81–2.57	0.209
**Management responsibility (ref: yes)**												
No	0.44	0.35–0.54	< 0.001	0.34	0.23–0.48	< 0.001	0.48	0.33–0.69	< 0.001	0.50	0.34–0.72	< 0.001
**Income level (ref: low)**												
Moderate	0.77	0.65–0.92	0.003	0.91	0.68–1.22	0.530	0.68	0.50–0.93	0.015	0.76	0.56–1.03	0.078
High	1.08	0.67–1.72	0.761	1.12	0.45–2.79	0.809	1.05	0.50–2.19	0.907	1.10	0.46–2.61	0.836
**Working hours per week (ref: ≤ 40)**												
> 40	1.89	1.61–2.23	< 0.001	2.08	1.56–2.78	< 0.001	1.61	1.21–2.14	0.001	2.00	1.51–2.66	< 0.001
**Working overtime (ref: never)**												
Occasion	2.50	1.86–3.35	< 0.001	1.98	1.24–3.19	0.005	2.76	1.66–4.61	< 0.001	2.91	1.63–5.18	< 0.001
Frequent	6.52	4.76–8.93	< 0.001	4.25	2.57–7.03	< 0.001	7.90	4.55–13.73	< 0.001	8.89	4.80–16.47	< 0.001
**Occupational development opportunities (ref: low)**												
Moderate	0.79	0.67–0.92	0.002	0.91	0.71–1.18	0.491	0.81	0.61–1.07	0.131	0.68	0.52–0.89	0.005
High	0.94	0.70–1.26	0.680	1.31	0.82–2.09	0.261	0.53	0.31–0.91	0.022	1.22	0.68–2.19	0.505

*Abbreviations:CI* confidence interval, *GPs* general practitioners, *OR* odds ratio, *ref* reference

^a^Adjustment for region (eastern China, central China, western China), age (21–, 30–, 40–, 50–), gender (male, female), marital status (unmarried/widowed/divorced, married), education level (associate’s degree or vocational diploma, bachelor degree, master degree or higher), work tenure (1–, 10–, 20–), contract status (permanent, temporary), professional title (elementary or below, intermediate, senior), management responsibility (yes, no), income level (low, moderate, high), working hours per week (≤ 40, > 40), working overtime (never, occasion, frequent), and occupational development opportunities (low, moderate, high)

^b^Adjustment for age (21–, 30–, 40–, 50–), gender (male, female), marital status (unmarried/widowed/divorced, married), education level (associate’s degree or vocational diploma, bachelor degree, master degree or higher), work tenure (1–, 10–, 20–), contract status (permanent, temporary), professional title (elementary or below, intermediate, senior), management responsibility (yes, no), income level (low, moderate, high), working hours per week (≤ 40, > 40), working overtime (never, occasion, frequent), and occupational development opportunities (low, moderate, high)

The results of stratified ordinal logistic regressions show that, for eastern Chinese GPs, professional title, management responsibility, working hours per week, and working overtime were predictors of occupational stress. For central Chinese GPs, gender, management responsibility, income level, working hours per week, working overtime, and occupational development opportunities were associated with occupational stress. Determinants for western Chinese GPs were age, contract status, management responsibility, working hours per week, working overtime, and occupational development opportunities.

## Discussion

This study found that the percentage of GPs who had a low, medium, and high level of occupational stress was 9.67%, 31.77%, and 58.56%, respectively. Most GPs reported that economic pressure was the main source of their occupational stress. The determinants of occupational stress were region, contract status, management responsibility, income level, working hours per week, working overtime, and occupational development opportunities. Regional variation in predictors of occupational stress among GPs was found in the stratified analysis.

The present study showed that more than half (58.56%) of Chinese GPs reported being very or extremely stressed at work, a relatively high prevalence when compared to that of GPs in developed countries (e.g., France, Germany, and the United States), which range from 18.0% to 58.9% [11]. However, it was lower than the prevalence found in some developing countries [e.g., Saudi Arabia (66.2%) [21] and Ethiopia (68.2%) [22]. This international variability may be explained by differences in sample size, study location, measurement tool, practice setting, socioeconomic status, culture, and healthcare systems.

Previous studies had investigated occupational stress among GPs using different measurement tools. The original or short form of the effort-reward imbalance questionnaire was commonly used in developed countries [12, 14, 15]. It was designed based on the effort-reward imbalance model and was validated in person-based service occupations (such as physicians and nurses). For measuring chronic stress, the Trier Inventory for the Assessment of Chronic Stress (TICS-SSCS) was a standardized and validated instrument [13, 17, 18]. This scale was used to measure strain contributing from chronic stress in the past three months and has been proved to be suitable for GPs. Lee et al. [19] developed the 20-item Family Physician Stress Inventory after conducting in-depth interviews with 10 family physicians. This questionnaire was focused on describing the strategies for coping with personal and occupational stress. In our study, we used one item to assess the prevalence of occupational stress among GPs, which was consistent with the Commonwealth Fund International Health Policy Survey of Primary Care Physicians [11]. It allowed for a comparison of occupational stress in our study population with the GPs in 11 high-income countries.

We found that the prevalence of occupational stress among Chinese GPs varied by region, though the ordinal logistic regression analysis indicated that not all such differences were statistically significant. GPs from eastern China had a 61.11% prevalence of occupational stress, ranking it first among all regions. Eastern China is the most densely populated and economically developed of the three regions. As such, millions of migrant workers enter the region each year [23]. One possible explanation of our regionally-varying findings is that the healthcare system in eastern China is becoming increasingly overloaded due to this migration, resulting in increased occupational stress within the health workforce [24].

Management responsibilities, working hours per week, and overtime work upon GPs’ occupational stress were consistent across regions. GPs with management responsibilities were more likely to report higher occupational stress, a pattern found elsewhere in the literature [11–13]. In many countries, excessive bureaucratization has led to increased regulatory and administrative responsibilities which limit the professional autonomy of health workers [15]. Lower levels of administrative autonomy were associated with higher occupational stress among primary care physicians in the US, the UK, and German health care systems [12]. A national study of 3,000 GPs conducted in the UK found that 80% of participants reported that they were asked to complete unimportant administrative duties, preventing them from completing more important duties [25]. In Germany, GPs are not only confronted with the challenges of patient care, but also must find time to complete a variety of administrative tasks [13]. More than half (54%) of German physicians complained about the amount of time they are required to spend completing administrative tasks [15]. Future research should investigate the deleterious effects of excessive administrative responsibilities.

Previous studies of occupational stress in GPs have identified time pressure and high workload as risk factors [11, 14]. The present study identified that working hours per week and working overtime were significantly associated with occupational stress in each region studied. According to a report from the Chinese Medical Doctors Association, 66.4% of doctors in the primary healthcare institution, including GPs, had worked more than 40 h per week and 66.67% of doctors had worked overtime in 2014 [26]. The figure for working more than 40 h per week in our study (41.84%) was quite different from the one reported in 2014 (66.4%). It may be explained by differences in sample size, study population, practice setting, and changes in the human resources of GPs between 2014 and 2017. Such work hours may lead to a gap between best evidence and clinical practice: 47.5% of doctors reported that working overtime left little time to participate in education and training activities [26]. Our finding that GP occupational stress was elevated among those working more than 40 h per week is evidence that long work hours may have an adverse effect on GPs in China. Overtime work may also have more direct adverse effects on medical practice, by causing fatigue that can detrimentally impact medical safety [8]. As a priority, efforts should be made to reduce the excessive demands placed upon GPs in China, but reform will require the joint action of GPs, their organizations, and the wider healthcare system.

In the stratified analyses, we found that the predictors of occupational stress were not consistent across regions. Occupational development opportunities were significantly associated with occupational stress for both central and western Chinese GPs, but this association was not observed in eastern China. Despite eastern China’s health system being strained by rapidly increasing demands, the region contains the greatest concentration of economic resources in China. As such GPs in the region typically have higher salaries and more occupational development opportunities [23]. In our study, the proportion of eastern Chinese GPs reporting moderate (44.43%) and high level of (9.36%) occupational development opportunities were greater than reported by central (39.24% and 6.08%, respectively) and western (40.73% and 6.08%, respectively) Chinese GPs, which was consistent with Chinese primary healthcare system status. Evidence suggests that many healthcare workers from less-developed regions of China choose to migrate to the more developed eastern regions [27]. This was one possible explanation for why no significant difference in occupational stress was found between levels of occupational development opportunities in eastern China.

We found that most GPs reported economic pressure as the main source of their occupational stress. Intriguingly, in the multivariable model, a statistically significant association between a moderate level of income and occupational stress was found in China, especially in central areas. However, no statistically significant association was found between high level of income and occupational stress. These findings may have been the result of hesitancy to share truthful income information among high-income GPs. Since the proportion of GPs reporting high income was relatively low in our study, this conclusion is plausible.

### Strengths and limitations

The present study investigates, for the first time, occupational stress and associated factors using a nationally representative sample of GPs in China. The study also had a large sample size, with 3,000 participants obtained from community health clinics in eastern, central, and western China. As such the study had good statistical power to detect determinates associated with occupational stress among Chinese GPs.

The reader should consider the limitations of our study. Firstly, it cannot establish causation because of its cross-sectional design. Secondly, it is possible that the self-reported data were subject to self-reporting bias. Nevertheless, most independent variables related to factual reports on lower-sensitivity personal characteristics, limiting the impact that social desirability bias (one form of self-reporting bias) may have on our results. Thirdly, one item may not exactly reflect the internal validity of occupational stress. Fourthly, some possible risk factors of occupational stress were not included in the analysis since no data were collected on them. Examples included having children, time spent commuting, work environment, or doctor-patient relationships. Future studies should be longitudinal and additionally investigate the potential predictors not included in our analysis. Occupational stress should be measured using standardized methods.

### Implications for research and practice

GPs are the gatekeepers of the health system, thus improvement in GP job satisfaction and GP retention can lead to health improvements population-wide. The present study provides important evidence to improve the management of the GP workforce in China. Our findings suggest that nationwide efforts to reduce occupational stress among Chinese GPs are best directed towards moderating work intensity, increasing opportunities for occupational development and increasing GP remuneration.

## Conclusions

In summary, Chinese GPs have a high level of occupational stress. Subnational region, contract status, management responsibility, income level, working hours per week, working overtime, and occupational development opportunities were significantly associated with occupational stress among Chinese GPs. Determinants of occupational stress varied by region. Strategies aiming to reduce Chinese GPs’ occupational stress must consider this regional variation.

## Data Availability

Data may be made available by contacting the corresponding author.

## Abbreviations

CHCs: Community health centers
CI: Confidence interval
GPs: General practitioners
OR: Odds ratio
SD: Standard deviation
